# The mycobacterial DNA-binding protein 1 (MDP1) from *Mycobacterium bovis *BCG influences various growth characteristics

**DOI:** 10.1186/1471-2180-8-91

**Published:** 2008-06-10

**Authors:** Astrid Lewin, Daniela Baus, Elisabeth Kamal, Fabienne Bon, Ralph Kunisch, Sven Maurischat, Michaela Adonopoulou, Katharina Eich

**Affiliations:** 1 Robert-Koch-Institut, Nordufer 20, 13353 Berlin, Germany; 2 Sanofi-Aventis Germany GmbH, TD Metabolism, 65926 Frankfurt am Main, Germany; 3 IUT de Dijon, Département Génie Biologique, Bd Dr Petitjean, 21078 Dijon Cedex, France; 4 Klinik für Allgemein-, Visceral- und Transplantations-Chirurgie, Charité-Campus Virchow, Augustenburger Platz 1, 13353 Berlin, Germany

## Abstract

**Background:**

Pathogenic mycobacteria such as *M. tuberculosis*, *M. bovis *or *M. leprae *are characterised by their extremely slow growth rate which plays an important role in mycobacterial virulence and eradication of the bacteria. Various limiting factors influence the generation time of mycobacteria, and the mycobacterial DNA-binding protein 1 (MDP1) has also been implicated in growth regulation. Our strategy to investigate the role of MDP1 in mycobacterial growth consisted in the generation and characterisation of a *M. bovis *BCG derivative expressing a MDP1-antisense gene.

**Results:**

The expression rate of the MDP1 protein in the recombinant *M. bovis *BCG containing the MDP1-antisense plasmid was reduced by about 50% compared to the reference strain *M. bovis *BCG containing the empty vector. In comparison to this reference strain, the recombinant *M. bovis *BCG grew faster in broth culture and reached higher cell masses in stationary phase. Likewise its intracellular growth in mouse and human macrophages was ameliorated. Bacterial clumping in broth culture was reduced by the antisense plasmid. The antisense plasmid increased the susceptibility of the bacteria towards Ampicillin. 2-D protein gels of bacteria maintained under oxygen-poor conditions demonstrated a reduction in the number and the intensity of many protein spots in the antisense strain compared to the reference strain.

**Conclusion:**

The MDP1 protein has a major impact on various growth characteristics of *M. bovis *BCG. It plays an important role in virulence-related traits such as aggregate formation and intracellular multiplication. Its impact on the protein expression in a low-oxygen atmosphere indicates a role in the adaptation to the hypoxic conditions present in the granuloma.

## Background

More than 120 years have passed since Robert Koch discovered that the bacterium *Mycobacterium tuberculosis *was the causative agent of tuberculosis. Since then enormous efforts have been undertaken to combat this disease. Despite of a number of achievements, *M. tuberculosis *still kills more people worldwide than any other bacterium, and the mechanisms transforming *M. tuberculosis *to the most successful bacterial pathogen are still not well understood. *M. tuberculosis *is able to multiply inside macrophages and to persist for decades in a latent state in the human host without being eliminated by the immune system. Our research aims at finding bacterial factors influencing intracellular survival and latency. Since the MDP1 gene (mycobacterial DNA-binding protein 1 gene) has been associated with the induction of a dormant state of mycobacteria [1], we decided to investigate the influence of MDP1 on *in vitro *growth, intracellular survival, antibiotic susceptibility and gene regulation.

MDP1 from *M. bovis *was first described in 1999 [2] and belongs to a group of orthologous DNA-binding proteins (Hlp, histone-like proteins) also present in other mycobacteria like *M. tuberculosis*, *M. kansasii*, *M. avium*, *M. fortuitum*, *M. marinum*, *M. leprae*, *M. ulcerans *or *M. smegmatis *[1]. MDP1 is composed of 205 amino acids, has a calculated molecular weight of 21 kDa and an isoelectric point of 12.4. The protein is rich in alanine, arginine, lysine, proline and threonine. MDP1 has partial homology with eukaryotic histone H1 and *Escherichia coli *HU protein, pointing to a possible role in DNA packaging and transcriptional regulation.

The MDP1 gene from *M. bovis *BCG is a single copy gene located between the genes *leu*D (isopropylmalate isomerase small subunit) and *mut*T1 (putative hydrolase). The genome of *M. bovis *BCG carries three other genes with low similarity to the C-terminal region of MDP1, encoding the 50S ribosomal protein L22, the heparin binding hemagglutinin (HBHA) and the histone-like protein HNS. The Hlp proteins from other slow-growing mycobacteria display 95% (*M. tuberculosis *strain H37Rv and *M. bovis *subsp. *bovis *strain AF2122/97), 84% (*M. ulcerans *isolate ITM), 81% (*M. leprae *strain TN) and 77% (*M. avium *strain 104) identical base pairs. The variations in the nucleotide sequences of the *hlp *genes from different mycobacterial species have been utilized for the establishment of specific PCR assays for diagnostic purposes [3-5].

It has recently been suggested that MDP1 was involved in growth regulation by controlling glycolipid biosynthesis and cell wall biogenesis [6]. MDP1 is a very abundant protein constituting 8–10% of the total protein. The MDP1 gene is up-regulated in stationary phase [2]. A correlation between the regulation of the MDP1 gene and the iron availability has recently been demonstrated. The protein Irep-28 (iron-regulated protein), which corresponds to the DNA-binding protein from *M. tuberculosis*, was shown to be up-regulated under low-iron conditions [7]. The MDP1 protein could be localized in the nucleoid, at the 50S ribosomal subunits and on the cell surface [2]. The surface localization of MDP1 and its capacity to bind hyaluronic acids, heparin and chondroitin sulphate imply a possible function as adhesion molecule facilitating entry into epithelial cells [8]. The protein ML-LBP21 from *M. leprae*, which is orthologous to MDP1, has been shown to be involved in the attachment to and invasion of Schwann cells [9].

The MDP1 gene from *M. bovis *BCG and the orthologous genes from *M. smegmatis *and *M. leprae *were mutated and the effects of the mutations on growth and adhesion were analyzed. Unexpectedly, the *hlp *mutant from *M. smegmatis *had the same generation time as the wild type and the mutation did not affect the viability of dormant cultures [10]. Similarly, the mutation of the MDP1 gene from *M. bovis *BCG did not affect the growth rate [11]. Analogous to these results, the capacity of *M. leprae *to bind to Schwann cells was not decreased after mutation of the *hlp *gene [12]. These results indicate that either the functions of the *hlp *genes are different from those assumed or that other genes replace the function of the *hlp *genes in the mutants.

Taking these results into account, we decided to use an alternative approach for the analysis of possible functions of MDP1 from *M. bovis *BCG. Here we describe the construction of a *M. bovis *BCG strain carrying a MDP1-antisense plasmid and the influence of the antisense construct on the growth of *M. bovis *BCG under various growth conditions.

## Methods

### Study design

We analysed the influence of the MDP1 protein on the growth characteristics of *M. bovis *BCG by reducing its expression *in M. bovis *BCG by means of a MDP1-antisense plasmid (pAS-MDP1). Two independent transformants containing the antisense construct [BCG(pAS-MDP1)] were generated to be able to rule out effects caused by second-line mutations. The empty cloning vector pMV261 was also introduced into *M. bovis *BCG, and the transformant BCG(pMV261) served as reference strain. The degree of MDP1 reduction in BCG(pAS-MDP1) was determined by ELISA. The characterisation of the phenotype of BCG(pAS-MDP1) in comparison to BCG(pMV261) comprised the determination of the growth rate in broth as well as in macrophages, the description of the degree of aggregate formation, the measurement of the susceptibility towards certain antibiotics and the analysis of proteomes of bacteria cultivated in different atmospheric oxygen concentrations.

### Construction of a *M. bovis *BCG derivative containing the MDP1-antisense plasmid pAS-MDP1

We amplified a 113 bp fragment of BCG-DNA, covering the first 102 bp of the coding sequence from the MDP1 gene and 11 bp of the untranslated upstream region containing the Shine-Dalgarno Sequence. Blast analyses with the sequence of the amplified fragment supplied evidence that this fragment has only negligible homology to other genes from BCG such that the probability of unspecific binding of the antisense fragment in BCG was extremely low. The PCR primers used (MDAS11, MDAS2; Table 1) were provided with restriction sites for the restriction enzymes HindIII (MDAS11) or PstI (MDAS2) to allow insertion of the amplified DNA into the HindIII/PstI-digested vector pMV261. This procedure resulted in an insertion of the beginning of the MDP1 gene in antisense orientation with respect to the *hsp*60 promoter (heat shock protein 60 promoter). Sequencing of the insert of the recombinant plasmid pAS-MDP1 confirmed the antisense orientation and the absence of mismatches. The plasmid pAS-MDP1 as well as the empty vector pMV261 were introduced into *M. bovis *BCG Copenhagen by electroporation. Experimental proof of transformation of the plasmids in BCG was achieved by PCR using the primers Tn903/S1 and Tn903/AS1 (specific for the *aph *gene from the vector pMV261; Table 1) and the primers MDAS1 and pMV261FW (specific for the antisense construct; Table 1). Expression of the antisense-RNA was proven by RT-PCR.

**Table 1:** Primers and probe used in this study.

**Gene**	**Primer pair, probe**	**Sequences of primers and probes^a^**	**References accession no.**
MDP1	MDAS11 MDAS2	*GGGAAGCTT*CGGAGGGTTGGGATGAACAAAGCAGA *GTGCTGCAG*ACGCACAATCGTGTCAACGACATT	GenBank: AB013441
*aph*	Tn903/S1 Tn903/AS1	CGA GGC CGC GAT TAA ATT CCA AC TGA GTG ACG ACT GAA TCC GGT GAG A	GenBank: DQ115380
MDP1 antisense construct	MDAS1 pMV261FW	GAA TTC GGA GGG TTG GGA TGA ACA AAG CAG A GAG GAA TCA CTT CGC AAT GGC	This study
85B antigen	MY85FW MY85BW 85Bprobe	TCAGGGGATGGGGCCTAG GCTTGGGGATCTGCTGCGTA (FAM)-TCGAGTGACCCGGCATGGGAGCGT-(TAMRA)	[20] [21]

^a^Restriction sites added to the primers for cloning purposes are italicised and underlined.

### Bacterial strains, plasmids, cell lines and growth conditions

The cultivation of *Mycobacterium bovis *BCG Copenhagen and *Escherichia coli *strain DH5α has been described before [13]. Media for growth of *M. bovis *BCG containing the plasmid pMV261 or pAS-MDP1 were supplemented with 25 to 50 μg Kanamycin ml^-1^. Plasmid pMV261 is an *E. coli*/*Mycobacterium *shuttle vector containing the Tn903-derived *aph *gene that confers Kanamycin resistance as a selectable marker and the promoter from the *hsp60 *gene for expression of integrated genes [14]. The mouse macrophage cell line J774A.1 (DSMZ no. ACC170) and the human monocyte cell line Mono-Mac-6 (MM6, DSMZ no. ACC124) were maintained in RPMI medium with 10% FCS.

### DNA and RNA manipulations

Molecular biology techniques were carried out according to standard protocols [15] or according to the recommendations of the manufacturers of kits and enzymes. PCR was performed with the PCR kit from MBI Fermentas. Oligonucleotide primers were purchased from Metabion. Restriction enzymes were obtained from MBI Fermentas and Biolabs. We used the QIAquick^® ^Gel Extraction kit (Qiagen) to elute DNA fragments from agarose gels. Purification of DNA samples to remove enzymes, nucleotides or salts was achieved either by phenol/chloroform extraction followed by alcohol precipitation or by using the QIAquick^® ^PCR Purification kit (Qiagen). Ligation reactions were carried out with the T4-DNA-Ligase from Biolabs or MBI Fermentas. Transformation of *E. coli *was performed according to the method by Hanahan [16]. *M. bovis *BCG was transformed by electroporation as previously described [13]. Plasmids were isolated from *E. coli *with the QIAGEN^® ^Plasmid Maxi kit (Qiagen) or with the NucleoSpin^® ^Plasmid kit from Machery-Nagel. Sequencing reactions were carried out by using the Prism Big Dye™ FS Terminator Cycle Sequencing Ready Reaction Kit from PE Applied Biosystems. QIAzol (Qiagen) was used to extract RNA from mycobacterial cultures. RT-PCR was performed with the Access RT-PCR System from Promega.

### Protein isolation

Proteins from mycobacterial broth cultures were prepared according to the method of Florczyk et al. [17] with modifications. Ten ml of bacterial cultures were sedimented and the pellets were washed three times with cold DPBS (10 mM sodium phosphate, 126 mM NaCl, pH 7.2) containing 0.2% EDTA. After addition of 1 μg ml^-1 ^of proteinase inhibitor cocktail (PIC, 1 mg ml^-1^, Sigma) the cells were heated for 30 min at 80°C and centrifuged. The pellets were resuspended in 200 μl Tris-SDS buffer (0.3% SDS, 50 mM Tris-HCl, pH 8) and the cells were disrupted in the cell disruptor Precellys 24 (PeqLab) by shaking them twice at 6000 rpm for 26 sec with a pause of 30 sec. The lysates were briefly centrifuged and SDS (final concentration: 2%) and β-mercaptoethanol (final concentration 5%) were added. The lysates were then incubated for 15 min at 37°C. The proteins were precipitated with acetone and resuspended in 100 to 1000 μl Tris-SDS buffer. The protein concentration was determined with the BCA Protein Assay (Pierce).

### MDP1 Elisa

Detection and quantification of MDP1 in protein preparations from broth cultures from *M. bovis *BCG(pAS-MDP1) and *M. bovis *BCG(pMV261) was achieved by ELISA, using a rabbit antiserum directed against the peptide 8 to 21 (DVLTQKLGSDRRQA) from MDP1 [GenBank:AB013441]. The peptide and antiserum were purchased from BioGenes GmbH. Beforehand the specificity of the antibody was confirmed by Western blotting using the BM Chemiluminescence Western blotting kit (Roche Diagnostics GmbH). For Western blotting, the MDP1 antiserum was diluted 1:3000. The luminescence signal was recorded with the chemiluminescence imager ChemieSmart 3000 (Vilber Lourmat).

The proteins isolated from the broth cultures were diluted in 50 mM NaHCO_3_, pH 9.6, and 100 μl of selected dilutions were given into the wells of NUNC-Immuno™ Maxisorp microtiter plates (Nalgene Nunc International) and incubated at 4°C over night. The wells were then washed twice with TBS-T (50 mM Tris-HCl, pH 7.8, 150 mM NaCl, 1 mM MgCl_2_, 0.05% Tween 80). Blocking of unspecific binding sites was achieved by incubation with 0.5% Casein in PBS for one hour at 37°C followed by five washes with TBS-T. Then, 100 μl of a 1:1500 dilution of the MDP1 antiserum or of pre-immune serum in TBS were added to the wells and incubated for 90 min at room temperature. After five washes with TBS-T 0.01 μg of the second antibody [Peroxidase-conjugated AffiniPure F (ab') 2 Fragment goat Anti-Rabbit IgG (H+L) (Jackson Immuno Research), diluted 1:7500 in TBS] was given into the wells and incubated for 90 min. The plate was washed again five times with TBS-T, and 100 μl SureBlue™ TMB Microwell Peroxidase Substrate (KPL) were added. The colour reaction was stopped by addition of 100 μl 1 M HCl and the absorption at 450 nm was measured with the Spectra Fluor (Tecan).

### Two-dimensional protein gels

About 200 μg of precipitated protein were resuspended and rehydrated in 450 μl rehydration buffer [8 M urea, 0.5% CHAPS (Roth), 0.2% DTT, 0.5% Pharmalyte (Amersham Biosciences), 0.002% bromphenol blue] for six hours. The rehydrated samples were transferred into strip holders together with IPG strips pH 3–11 NL (non-linear), 24 cm (Amersham Biosciences). The strips were covered with 500 μl cover fluid and focused on an Ettan IPGphor II unit (Amersham Biosciences) according to the manufacturer's instructions. In the second dimension, the proteins were separated in a vertical 12.5% SDS-polyacrylamide gel using the Ettan Daltsix electrophoresis unit (Amersham Biosciences). The gels were silver-stained according to the protocol by Blum et al. [18].

### Growth experiments in broth culture

Comparison of the growth rates of *M. bovis *BCG(pAS-MDP1) and *M. bovis *BCG(pMV261) was carried out by inoculating Middlebrook 7H9 medium containing 25 μg ml^-1 ^Kanamycin to obtain an initial optical density (OD) (600 nm) of 0.02 to 0.04 and measuring the ODs of the cultures at least twice per week. Growth of the strains was monitored by quantification of the ATP content of the cultures with the luminescence-based kit BacTiter-Glo™ Microbial Cell Viability Assay (Promega). The luminescence was reported as relative light units (RLU) with the microplate luminometer LB96V (EG&G Berthold).

The stability of mycobacterial aggregates in broth was examined by sonication in the Sonifier Cell disruptor W-450 (Branson Ultrasonic Corporation) at 20 watt with 50% pulse duration (pulse duration 30 sec min^-1^) for two to 20 minutes, followed by plating of appropriate dilutions on Mycobacteria 7H11 agar.

Growth of the bacteria under microaerobic (6.2 to 13.2% oxygen) or anaerobic (less than 0.1% oxygen) conditions was achieved by cultivation in the GENbox system from BioMérieux.

### Cell culture experiments

Infection of the macrophage cell line J774A.1 with *M. bovis *BCG strains was performed according to the protocol described previously [13,19]. A total of 5 × 10^4 ^cells of J774A.1 per well were infected at a multiplicity of infection (MOI) of five for four hours with *M. bovis *BCG(pAS-MDP1) or *M. bovis *BCG(pMV261) grown to OD 2.0 (600 nm).

The MM6 cells growing non-adherently were initially infected in 25 cm^2 ^cell culture flasks (Biochrom). 5 × 10^6 ^MM6 cells in 5 ml of RPMI medium were infected at a MOI of 20 for four hours with cultures of the *M. bovis *BCG strains grown to OD 2.0 (600 nm). The cells were then washed twice with 30 ml of RPMI and treated with Amikacin (200 μg ml^-1^) for two hours. After Amikacin treatment the infected MM6 were washed again, resuspended in RPMI medium with 5 μg ml^-1 ^of Amikacin, counted and distributed into 24-well plates (1 × 10^5 ^cells ml^-1 ^per well).

The extraction of DNA from the cell lysates was performed as described before [19]. Quantification of intracellularly grown BCG by real-time PCR was achieved by amplification of a region of the 85B antigen gene, using the primers MY85FW and MY85BW [20] (Table 1) together with the FAM/TAMRA-labelled probe My85Bprobe [21] (Table 1) as described [19]. The amount of DNA was determined by means of a standard established with known amounts of genomic DNA from *M. bovis *BCG.

## Results

### The antisense plasmid pAS-MDP1 reduces the expression of MDP1 in *M. bovis *BCG by about 50%

**Figure 1:**
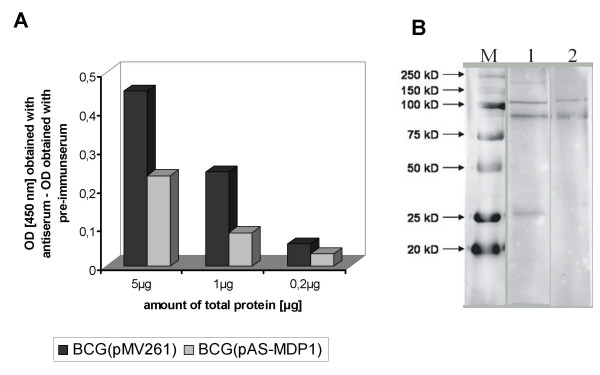
**Quantification of MDP1 by ELISA**. Proteins were extracted from broth cultures of the strains *M. bovis *BCG(pAS-MDP1) and *M. bovis *BCG(pMV261) grown until OD (600 nm) of 2.0. (A) 5, 1 and 0.2 μg of total protein were loaded into Microwell plates and incubated with either a MDP1-specific antiserum or with pre-immune serum. The OD at 450 nm reflects the amount of MDP1 antibody bound by the MDP1 protein. The diagram shows the values obtained after subtraction of the ODs measured with the pre-immune serum from the ODs measured with the MDP1 antiserum. (B) 30 μg of total protein from BCG(pMV261) were separated in a 10% SDS-PAGE, transferred onto a PVDV membrane and incubated either with MDP1 antiserum (1) or with the corresponding pre-immune serum (2). M: protein marker.

After electroporation of the plasmid pAS-MDP1 and the vector pMV261 into *M. bovis *BCG, we analyzed the impact of the antisense construct on the expression of MDP1 by ELISA using a MDP1-specific peptide antibody. The specificity of the antibody had first been confirmed by Western blotting. The Western blot was performed with a protein preparation from *M. bovis *BCG(pMV261) using either the MDP1 antiserum or the corresponding pre-immune serum. As shown in Fig. 1B, a protein larger than 25 kDa appeared specifically only if the MDP1 antibody was employed. This result is in line with the observation from Matsumoto and colleagues [2], who identified MDP1 as a 28 kDa protein in SDS-PAGE, although its molecular weight was calculated to be 21 kDa.

The MDP1 amount was measured by ELISA in cultures of *M. bovis *BCG(pAS-MDP1) and *M. bovis *BCG(pMV261) grown to OD 2.0 (600 nm). As shown in Figure 1A, the antisense plasmid pAS-MDP1 caused a considerable reduction of the expression of MDP1. On average, *M. bovis *BCG(pAS-MDP1) synthesized about half the amount of MDP1 that was produced by *M. bovis *BCG(pMV261).

### MDP1 regulates the growth of *M. bovis *BCG in broth culture

**Figure 2:**
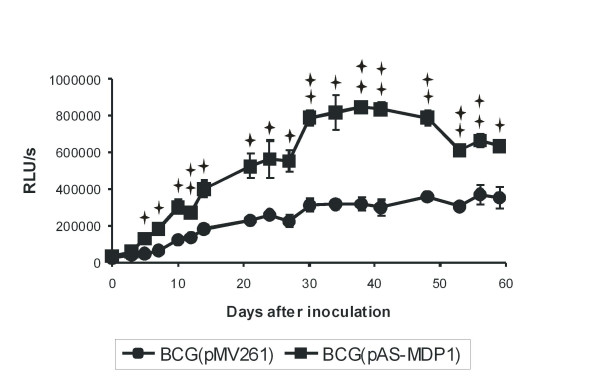
**Growth in broth culture**. Broth cultures from *M. bovis *BCG(pAS-MDP1) and *M. bovis *BCG(pMV261) were diluted into Middlebrook 7H9 broth to give an initial OD (600 nm) of 0.02 to 0.04 and the cultures were incubated during 59 days. The growth curves of the strains were generated by determination of the ATP content [displayed as relative light units (RLU)] in the cultures with a luciferase assay. The values represent the mean of three independent cultures with the standard deviation. The single asterisks indicate values that varied significantly (P < 0.05) and the double asterisks indicate values that varied very significantly (P < 0.01) according to the student's *t *test.

The influence of the reduction of the amount of MDP1 protein on the *in vitro *growth rates of *M. bovis *BCG was determined by comparing growth curves of broth cultures from *M. bovis *BCG(pMV261) and *M. bovis *BCG(pAS-MDP1) generated by measurement of the OD of the cultures (data not shown) and their ATP content (Figure 2). The antisense construct not only accelerated the growth of BCG but in addition enabled the bacteria to achieve a higher cell density in the stationary growth phase. The RLU values obtained with stationary phase cultures from *M. bovis *BCG(pAS-MDP1) exceeded the RLU values obtained with cultures from *M. bovis *BCG(pMV261) by factors of up to about 2.5 (Figure 2). We wondered whether the growth curves generated by measurement of the ATP content of the cultures might have been influenced by different ATP synthesis rates of the two strains. Therefore, we also measured the DNA content of the cultures by quantitative real-time PCR. The measurement of the growth rates of the two strains by DNA quantification also demonstrated a faster growth of the *M. bovis *BCG(pAS-MDP1) and its ability to achieve a higher cell mass in stationary phase (data not shown).

### MDP1 influences the aggregation of *M. bovis *BCG

**Figure 3:**
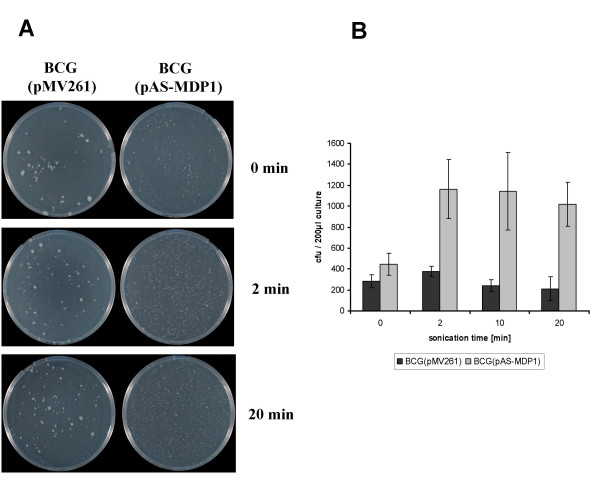
**Aggregate formation**. Broth cultures from *M. bovis *BCG(pAS-MDP1) and *M. bovis *BCG(pMV261) were diluted to an OD (600 nm) of 0.02 to 0.04 and 200 μl were either directly plated on agar plates (0 min) or plated after having been sonified for 2 min to 20 min. The incubation time after plating was three weeks. Panel (A) shows the colonies and in panel (B) the colony numbers are displayed. The columns in (B) represent the mean of 5 plates with the standard deviation.

Cell aggregation of *M. bovis *BCG(pMV261) and *M. bovis *BCG(pAS-MDP1) was visualized by growing broth cultures of both strains and plating dilutions on agar plates. As shown in Figure 3A, the colonies formed by the strain containing the vector pMV261 were very variable in size, pointing to the formation of large irregular aggregates in the broth culture. On the other hand, the strain containing the antisense construct formed more regularly sized and smaller colonies, indicating a less pronounced clumping of the bacteria in broth.

The stability of the aggregates generated by the two strains was investigated by analysis of the efficiency of aggregate disruption by sonication of diluted broth cultures for different time periods and plating of the sonified cultures on agar plates. Figure 3A and 3B demonstrate the reduced stability of aggregates from *M. bovis *BCG(pAS-MDP1) compared to *M. bovis *BCG(pMV261). A sonication for two minutes sufficed to disrupt to a large extent the aggregates formed by *M. bovis *BCG(pAS-MDP1) as was evident from the appearance of an enhanced number of colonies (Figure 3B). The culture of *M. bovis *BCG(pMV261), on the other hand, contained very large aggregates even after having been sonified for 20 minutes. The sonified culture still generated very large and variable colonies and only few small colonies were visible. The colony number of BCG(pMV261) had not augmented compared to the culture that had not been sonified (Figure 3B).

### MDP1 has an impact on the susceptibility of *M. bovis *BCG to Ampicillin

**Figure 4:**
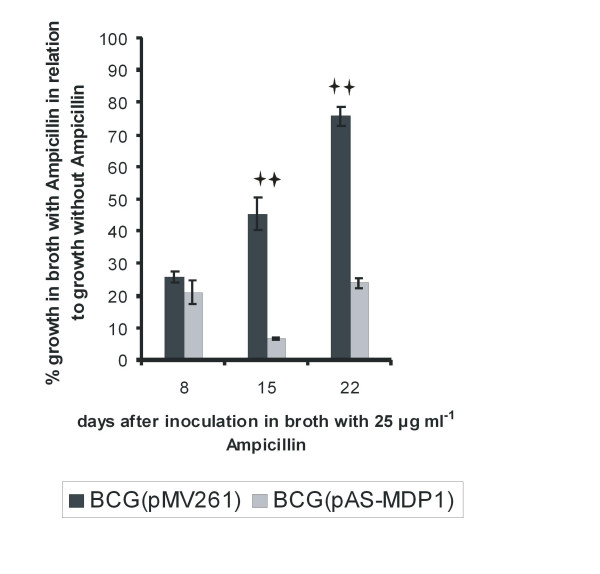
**Susceptibility towards Ampicillin**. Broth cultures from *M. bovis *BCG(pAS-MDP1) and *M. bovis *BCG(pMV261) were diluted into Middlebrook 7H9 broth without or with Ampicillin (25 μg ml^-1^) to give an initial OD (600) nm of 0.02 to 0.04. The growth of the strains was monitored by determination of the ATP content with a luciferase assay during 22 days. The diagram displays the percent growth of the strains in medium with Ampicillin in relation to the growth in medium without Ampicillin. The columns represent the mean of three independent cultures with the standard deviation. The double asterisks indicate values that varied very significantly (P < 0.01) according to the student's *t *test.

Changes in growth characteristics of bacteria may also influence their susceptibility towards antibiotics. To test this possibility, we measured the inhibitory effect of two selected antibiotics on the growth of *M. bovis *BCG(pMV261) and *M. bovis *BCG(pAS-MDP1). Rifampicin was chosen because of its importance for tuberculosis therapy. Ampicillin was selected because MDP1 is a cell wall-associated protein and has been shown to be involved in cell wall biogenesis [6]. We inoculated cultures of the two strains in Middlebrook medium without antibiotics as well as in medium with antibiotics and measured their growth during 22 days by quantification of the ATP content of the cultures with a luciferase assay. The growth rates were displayed as relative light units (RLU). We then calculated the relative growth at a given time point by setting the RLU values achieved in cultures without antibiotics to 100%. The antisense construct did not significantly change the susceptibility towards Rifampicin (data not shown). However, it had a strong effect on the susceptibility towards Ampicillin (Figure 4). After 15 and 22 days of growth in medium containing 25 μg ml^-1 ^Ampicillin, the *M. bovis *BCG containing pAS-MDP1 was much more affected by the presence of the antibiotic than the reference strain. The most pronounced difference occurred after 15 days. At this time point, the BCG strain with the antisense-plasmid displayed a relative growth rate in the presence of Ampicillin of only 6.5%, while the BCG with pMV261 displayed a relative growth rate of 45%.

### MDP1 affects the protein expression of *M. bovis *BCG grown in low oxygen conditions

**Figure 5:**
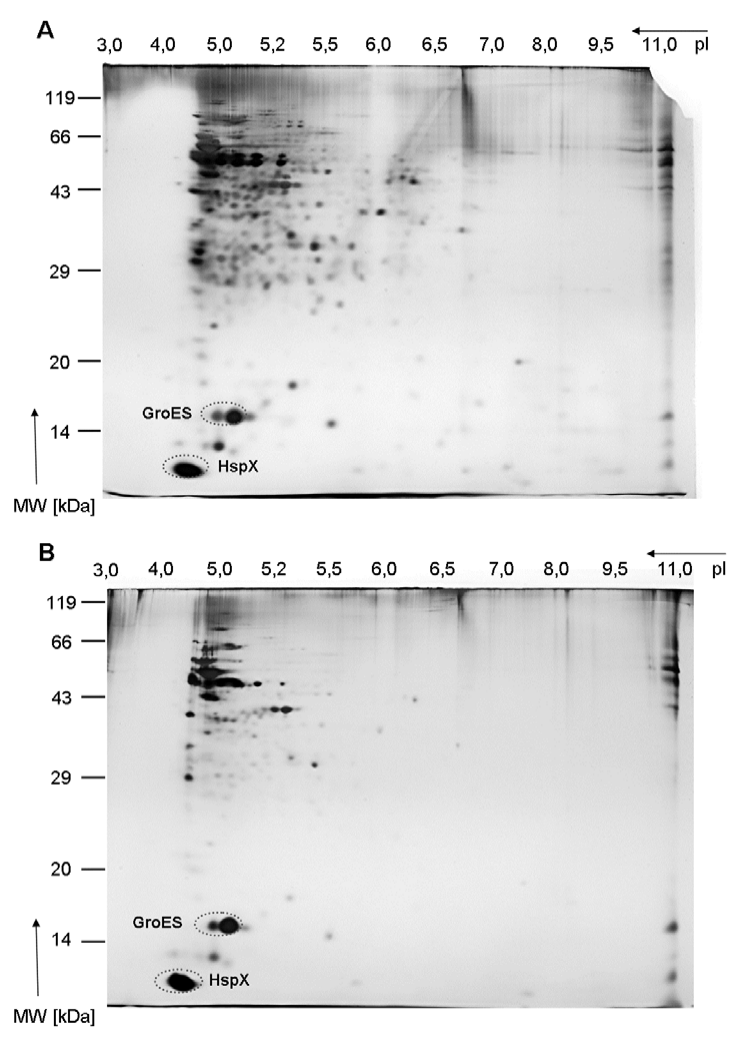
**Protein patterns of microaerobically grown cultures**. *M. bovis *BCG(pMV261) (A) and *M. bovis *BCG(pAS-MDP1) (B) were grown under microaerobic conditions during ten days, and total protein was isolated. 200 μg of protein were first separated by isoelectric focussing according to their pI and then in 12.5% SDS-polyacrylamid gels according to their size. The circles mark the spots containing the proteins GroES and HspX.

The influence of MDP1 on gene regulation was analysed by generation of 2-dimensional protein gels. Proteins were isolated both from cultures grown aerobically to an OD 2.0 (600 nm) and from cultures which were first grown aerobically to OD 2.0 and then transferred into a microaerobic atmosphere for ten days. The protein patterns of two strains grown aerobically did not diverge strongly (data not shown). However, if the cultures were maintained in an oxygen-poor atmosphere, the *M. bovis *BCG strain containing the antisense construct pAS-MDP1 showed a protein pattern that strongly differed from the reference strain containing the empty vector pMV261 (Figure 5). The proteome gels of the strain *M. bovis *BCG(pAS-MDP1) (Figure 5B) exhibited less protein spots than those of the strain *M. bovis *BCG(pMV261) (Figure 5A), and the spot intensity was also reduced for many of the proteins. Interestingly, the spots representing the stress-related proteins GroES and HspX were not affected by the presence of the antisense plasmid pointing to different effects of a reduction of the MDP1 amount on different proteins. On account of the extremely high isoelectric point (pI) of 12.4 of the MDP1 protein, it was not possible to identify MDP1 itself in the two-dimensional gels. Two-dimensional protein gels from cultures transferred from a microaerobic to an anaerobic atmosphere for 14 days showed protein patterns similar to those obtained with the protein preparations from microaerobically grown cultures (data not shown). The influence of the amount of MDP1 on the protein expression in cultures grown in microaerobic or anaerobic conditions indicates a role of MDP1 in the adaptation to oxygen limitation.

### MDP1 influences the persistence of *M. bovis *BCG in macrophages

**Figure 6:**
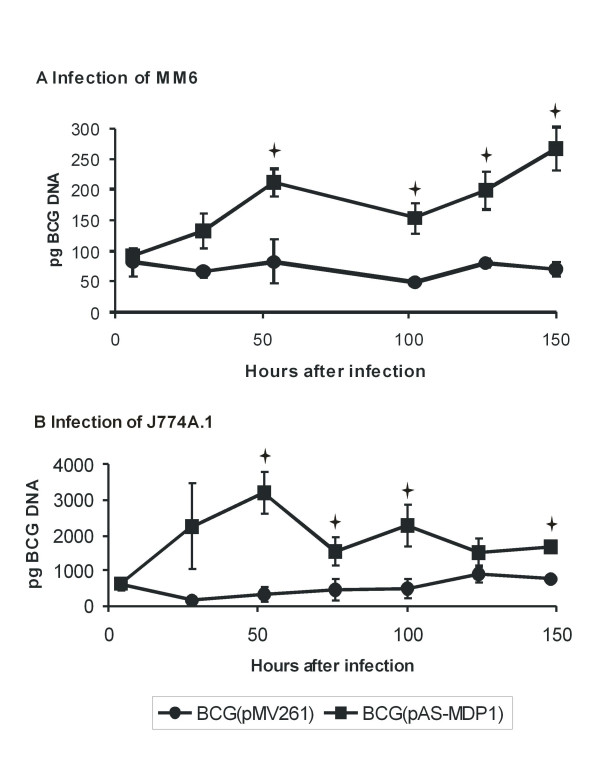
**Intracellular growth**. MM6 macrophages (A) and J774.A1 macrophages (B) were infected with *M. bovis *BCG(pAS-MDP1) and *M. bovis *BCG (pMV261). The DNA of intracellular bacteria was quantified by real-time PCR in 10 μl of a DNA preparation of lysed macrophages. The amounts of intracellular bacteria post infection are represented by the values at 6 h in part (A) and by the values at 4 h in part (B). The values represent the mean of three independent infections with the standard deviation. The single asterisks indicate values that varied significantly (P < 0.05) according to the student's *t *test.

One of the most important virulence-related features of highly pathogenic mycobacteria is their ability to survive and multiply in macrophages. We therefore wondered whether the amount of MDP1 had an influence on the survival of *M. bovis *BCG in mouse and human macrophage lines. Cultures of both strains grown to an OD 2.0 (600 nm) were used to infect the mouse macrophage line J774.A1 and the human macrophage line MM6. The amount of intracellular bacteria was monitored by quantification of the DNA content with real-time PCR during six days. As shown in Figure 6A and 6B, there was no difference in the initial infection rate of either *M. bovis *BCG(pMV261) or *M. bovis *BCG(pAS-MDP1). However, the antisense construct strongly enhanced the growth of *M. bovis *BCG within both macrophage lines. The amount of the BCG derivative containing the empty vector stayed relatively constant throughout the whole experiment. In contrast, the BCG with the antisense plasmid multiplied during the first two days after infection and had reduplicated in MM6 and even multiplied by six in J774.A1. After this the bacterial load of *M. bovis *BCG(pAS-MDP1) somewhat decreased in both cell lines and then stayed relatively constant in J774.A1 and even increased again in MM6.

## Discussion

The MDP1 protein belongs to a unique class of histone-like proteins (Hlp) specific for mycobacteria. Orthologous genes were identified in fast-growing apathogenic mycobacteria like *M. smegmatis *(*hupB *gene) [10] as well as in pathogenic species like *M. bovis *(MDP1 gene) [2], *M. tuberculosis *(*hlp *gene) [22], *M. leprae *(ML-LBP21-gene) [9], *M. avium *[23] and *M. paratuberculosis *[23]. The MDP1 protein possesses a striking combination of two features: it is a DNA-binding protein which is also surface exposed [2]. The Hlp have been associated with a large variety of cellular functions in different mycobacteria like replication, transcription and translation [24], growth regulation [1], adaptation to dormancy [10] and adhesion to Schwann and epithelial cells [8,9]. Recently, the MDP1 gene from *M. bovis *has been shown to play an important role in cell wall biosynthesis by binding to the antigen 85 (Ag85) and to its substrate trehalose-6-monomycolate [6]. The extremely strong expression of Hlp reaching up to 8–10% of the total protein amount in *M. bovis *BCG [2] suggests its high importance for mycobacterial growth and survival. This makes it all the more surprising that mutations in *hlp *genes had a relatively small effect on the phenotypes of the mutants. The disruption of the *hup*B gene from *M. smegmatis *neither changed the generation time nor the viability of dormant cultures in the study by Lee et al. [10]. Katsube et al. [6] also observed only a slight effect on growth rate by mutagenesis of the *hlp *gene from *M. smegmatis*. Similarly, a MDP1 knock-out mutant from *M. bovis *BCG showed no change in growth rate compared to the wild type BCG [11]. A mutation of the *M. leprae hlp *gene also did not affect the capacity to bind to Schwann cells [12]. Although these studies were performed either with other mycobacterial species or by using other testing methods, the discrepancy between the slight phenotypic changes caused by deletion mutagenesis or insertion mutagenesis compared to the strong changes caused by our antisense gene is astonishing. One possible explanation for the unexpected outcomes of the mutagenesis experiments is the complementation of the mutated *hlp *genes by other genes. For instance, it has been observed that the deletion of the main porin gene *mspA *from *M. smegmatis *entails an expression of otherwise silent porin genes [25]. A mycobacterial protein potentially able to fulfil some of the functions of Hlp is the heparin-binding hemagglutinin (HBHA). This protein exhibits striking similarities to Hlp. Both proteins produce a band at MW 28 kDa in SDS gels, are surface exposed and promote binding to epithelial cells [26]. They are both post-translationally methylated and show cross-reactivity due to the presence of copies of the motif APAKKAA [27]. According to studies of Verbelen et al. [28], HBHA is mainly responsible for mycobacterial aggregation.

Since mutagenesis of *hlp *genes had not provided much information on their function, we chose the antisense technique as alternative method to assess the importance of the MDP1 gene for the growth behaviour of *M. bovis *BCG. We hoped that reducing the amount of MDP1 by an antisense construct instead of completely removing the protein by mutagenesis would lessen complementation effects by activation of other genes with similar functions. The antisense-fragment was selected taking into consideration the recommendation that an efficient antisense-RNA should cover the start codon and the Shine-Dalgarno-Sequence and have a size of at least 5% of the gene length [29]. The antisense technique has contributed to the clarification of the function of many mycobacterial genes. Advantages of this technique are the possibility to analyse essential genes whose mutagenesis would be lethal and to repress genes present in several copies. Examples of the application of the antisense technique in mycobacteria are the repression of *ahpC *from *M. bovis *[30], *dnaA *from *M. smegmatis *[31], FAP-P from *M. avium *subsp. *paratuberculosis *[32] or *pknF *from *M. tuberculosis *[33]. The degree of repression by antisense plasmids was estimated to be 10 to 30% in the study of Secott et al. [32] and 65% in the work of Greendyke et al. [31]. Greendyke and colleagues achieved this relatively strong repression by using the same vector system (pMV261 with the *hsp*60 promoter) as the one employed in our study. We could demonstrate a reduction in the amount of MDP1 by the antisense construct of about 50% in cultures grown aerobically in Middlebrook 7H9 broth. Since use of the antisense technique does not allow the proof of the absence of second-line mutations by complementation, we generated two independent *M. bovis *BCG transformants containing the plasmid pAS-MDP1 and analysed their effect on the growth of BCG.

The influence of the antisense construct on the growth rate was shown to be considerable. The antisense strain not only grew faster than the reference strain, it also reached a much higher cell mass in stationary phase. The growth curves were generated by measurement of the ATP content, a method only considering living cells. We favoured this method over plating and counting of colony forming units (cfu), because we estimate it to be more appropriate for out test system employing standing cultures for the measurement of growth over a relatively long time period. It has been shown that a proportion of the bacteria present in stationary phase and sustaining anoxia are not able to form colonies if plated on agar, although they are metabolically active, generate steady ATP amounts and are able to grow in broth [34-36]. To exclude the possibility of varying ATP contents as a consequence of varying metabolism of the strains, we also quantified the bacteria by quantitative real-time PCR. The DNA quantification confirmed the faster growth of *M. bovis *BCG(pAS-MDP1) compared to the growth of the reference strain. This result is in contrast to the findings of Chen et al. [11], who observed no influence on the growth rate of their *M. bovis *BCG MDP1 mutant. However, it supports the hypothesis by Matsumoto and colleagues [1]. These authors transformed rapidly growing mycobacteria into slow growing bacteria by transferring the MDP1 gene into the fast growers and postulated a participation of MDP1 in growth regulation and induction of the dormant state. A participation in the induction of the dormant state is also supported by the achievement of much higher cell masses in stationary phase observed in our study. Under the influence of the antisense construct, the transition from the active growth phase into the stationary phase occurs at higher cell densities. This observation supports the hypothesis that up-regulation of MDP1 in stationary phase and dormant cultures results in a growth arrest which is delayed in the antisense strain expressing less MDP1 protein.

The MDP1 protein is surface exposed and has been shown to aggregate to nanomer polypeptides [2]. A lower amount of MDP1 on the surface of the bacteria may explain the reduced clumping and decreased stability of aggregates of *M. bovis *BCG(pAS-MDP1) compared to the reference strain. This finding implicates that the HBHA protein mainly held responsible for the formation of mycobacterial aggregates [28] is not the only factor mediating this characteristic feature of mycobacteria. The clumping of mycobacteria not only complicates many experimental procedures like determination of growth and infectious doses but also influences other important features. For example the clumping of *M. tuberculosis *is most pronounced in virulent strains [37], and clumping can influence the accessibility of antibiotics into the bacteria. The reduced clumping of the antisense strain may also have contributed to its better growth in broth culture by ameliorating the uptake of nutrients.

An influence of the antisense construct on the susceptibility of *M. bovis *BCG to antibiotics was tested for Rifampicin and Ampicillin (Figure 4). Rifampicin was selected because of its therapeutical importance. Ampicillin interferes with the cell wall synthesis and was chosen because MDP1 plays a role in cell wall synthesis as was proven by Katsube et al. [6]. While we could measure no significant effect of the antisense construct on the susceptibility towards Rifampicin, it induced a strong increase in the susceptibility towards Ampicillin. The interference of the antisense plasmid with the reaction against only one of the two antibiotics argues against a simply better diffusion of the antibiotics into the cells due to the reduced clumping of the bacteria, but suggests a more specific mechanism related to the mode of action of Ampicillin. Ampicillin inhibits the formation of cross-links in the peptidoglycan layer. The accelerated growth of *M. bovis *BCG(pAS-MDP1) requires a higher rate of peptidoglycan synthesis, thus perhaps rendering the bacteria more susceptible to Ampicillin. It is also possible that the influence of the reduction in the amount of MDP1 on glycolipid synthesis enhances the diffusion of Ampicillin through the bacterial cell wall. As a consequence the critical amount of Ampicillin, which cannot be inactivated by the mycobacterial β-lactamases, may be reached at lower Ampicillin concentrations than in the wild-type cells.

For further investigation of a possible involvement of MDP1 in the regulation of latency, we also performed growth experiments in microaerobic and anaerobic atmospheric conditions. In these experiments, cultures of the two strains were first maintained in an atmosphere containing 6.2% to 13.2% oxygen. In this way we simulated the situation in granulomas, where low-oxygen conditions are prevailing [38,39]. From these microaerobic conditions, the cultures were transferred into an anaerobic atmosphere with less than 0.1% oxygen. We could not find significant growth differences between the two strains under low-oxygen conditions. While the protein patterns of aerobically grown cultures of the two strains did not differ significantly, the proteome analysis of cultures grown under low-oxygen conditions revealed a much stronger effect of the oxygen limitation on the *M. bovis *BCG strain expressing less MDP1 compared to the reference strain. The number of visible protein spots as well as the intensity of many spots was reduced in the antisense strain. It can therefore be assumed that MDP1 plays a role in the adaptation of the bacteria to hypoxic conditions, which is a prerequisite for the survival in the granuloma.

The MDP1 protein has homology to the HU class (heat-unstable nucleoid protein) of DNA-binding proteins. HU proteins are capable of wrapping DNA and stabilizing it from denaturation in extreme environmental conditions. Together with H-NS (histone-like DNA structuring proteins), IHF (integration host factor), FIS (factor for inversion stimulation), LRP (leucine-responsive regulatory protein) and Dps (DNA-binding protein from starved cells) they belong to a group of proteins which bind relatively unspecific to DNA. Binding of theses proteins to DNA modulates the topology of the according DNA regions and thus their accessibility for proteins involved in transcription. Hereby the expression of genes lying in the concerned regions is affected [40,41]. The affinity of these DNA-binding proteins to the DNA is influenced by the environment, so that they are able to mediate the adaptation to changes in the environmental conditions. Further studies are required to find out whether MDP1 plays a role in adapting the gene expression pattern to the conditions occurring during the infection process.

*M. tuberculosis *has an intracellular lifestyle and we therefore tested whether the growth acceleration caused by the antisense construct and the achievement of higher bacterial loads observed in broth also occurred inside macrophages. We quantified intracellular BCG by real-time PCR instead of colony counting, since it has been found by other authors that a substantial proportion of intracellular mycobacteria released from macrophages was unculturable by cfu plating, although the presence of viable cells could be shown with the MPN (dilution to extinction in liquid medium) method [42]. Real-time PCR methods have also been evaluated for the quantification of other intracellular bacteria in macrophages, such as *Legionella pneumophila *[43]. Batoni et al. [44] had shown that the *hsp*60 promoter from *M. avium *is not only active in bacteria growing in broth cultures but also in mycobacteria present inside murine macrophages. We therefore assumed that a repression of MDP1 expression by the antisense plasmid was also given during intracellular growth of the mycobacteria. The MDP1 antisense construct caused an increase in the amount of mycobacterial DNA in both a human as well as a murine macrophage line. According to this, the MDP1 protein is involved in extra- as well as intracellular growth regulation. Higher bacterial loads can influence many responses of the infected cells, like the degree of macrophage stimulation, cytokine expression or triggering of apoptosis. The MDP1 protein may therefore play an important role for virulence of mycobacteria.

## Conclusion

The most striking feature of the MDP1 protein is its diversity with respect to localisation, biochemical properties and potential functions. The results obtained in this study are in line with several of its proposed functions. We observed a relatively strong effect on the extra- as well as intracellular growth rate, supporting the idea that MDP1 plays a role in the regulation of the generation time. The reduced aggregate formation of the antisense strain agrees with its localization on the cell surface of the bacteria and strengthens the hypothesis of a role in adhesion. The enhanced susceptibility of the antisense strain towards Ampicillin supports the theory of its function in cell wall biosynthesis. Finally, the diverging protein patterns of cultures of the antisense strain and the reference strain grown in low-oxygen atmosphere indicate a role in gene regulation and adaptation to dormancy and latency. Our study demonstrates that MDP1 fulfils various functions important for the infection of host cells and the maintenance of the infection within the granuloma. Our future efforts will concentrate on the analysis of the participation of MDP1 in granuloma formation and the persistence of mycobacteria inside these structures.

## Authors' contributions

AL designed and supervised the project, performed experiments to determine the antibiotic susceptibility of the strains and wrote the manuscript. DB constructed the antisense plasmid and one of the recombinant *M. bovis *BCG strains containing this plasmid. EK performed the growth experiments. FB constructed the second *M. bovis *BCG strain carrying the antisense plasmid and participated in the testing of the antibiotic susceptibility of the strains. RK and SM characterised the proteomes of the *M. bovis *BCG derivatives. MA performed the experiments describing the formation and stability of aggregates of the strains and tested the antibiotic susceptibility. KE performed the ELISA experiments.
